# Prevalence and socio-demographic correlates of psychological health problems in inhabitants of the West Pomeranian Voivodeship during the COVID-19 outbreak

**DOI:** 10.1192/j.eurpsy.2022.1239

**Published:** 2022-09-01

**Authors:** A. Cybulska, K. Rachubińska, D. Schneider-Matyka, E. Grochans

**Affiliations:** 1 Pomeranian Medical University, Department Of Nursing, Szczecin, Poland; 2 Pomeranian Medical University in Szczecin, Department Of Nursing, Szczecin, Poland

**Keywords:** Depression, Covid-19, Insomnia, Anxiety

## Abstract

**Introduction:**

Psychological health problems, especially emotional disorders, have become an important topic of considerations for many scientists, because the epidemiology of these disorders is strongly influenced by stressful events, such as the SARS-CoV-2 coronavirus pandemic.

**Objectives:**

The aim of this study was to evaluate selected parameters of psychosocial functioning as well as socio-demographic correlates of depression, anxiety, sleep disorders and perceived stress among the residents of the West Pomeranian Voivodeship.

**Methods:**

An online questionnaire was completed by 323 participants, in whom the parameters of psychosocial functioning were assessed: symptoms of depression (PHQ-9), anxiety (GAD-7), severity of sleep disorders (AIS) and perceived stress (Perceived Stress Scale).

**Results:**

The majority of the respondents (75.2%) scored high on the Perceived Stress Scale, almost half of the respondents (47.1%) had sleep disorders and 26% of the participants had no depressive symptoms. Age statistically significantly correlated with the severity of depressive symptoms and sleep disorders. Parental status statistically significantly correlated with the severity of depressive symptoms. There was a strong correlation between the severity of depression and anxiety and a strong correlation between depression and sleep disorders.

**Conclusions:**

Age and parental status contributed to the severity of depressive symptoms and the occurrence of sleep disorders among the residents of the West Pomeranian Voivodeship during the SARS-CoV-2 pandemic. Some of the residents of the West Pomeranian Voivodeship showed high levels of stress or insomnia. The severity of depressive symptoms significantly influenced the levels of anxiety and perceived stress, as well as the occurrence of sleep disorders.

**Disclosure:**

No significant relationships.

